# Influence of total polar compounds on lipid metabolism, oxidative stress and cytotoxicity in HepG2 cells

**DOI:** 10.1186/s12944-019-0980-0

**Published:** 2019-02-01

**Authors:** Jingjie Ju, Zhaojun Zheng, Yong-jiang Xu, Peirang Cao, Jingwei Li, Qiu Li, Yuanfa Liu

**Affiliations:** 1 School of Food Science and Technology, National Engineering Laboratory for Cereal Fermentation Technology, Collaborative Innovation Center of Food Safety and Quality Control in Jiangsu Province, Jiangnan University, 1800 Lihu Road, Wuxi, 214122 Jiangsu People’s Republic of China; 2 Shandong LuHua group co., LTD, Laiyang, 265200 People’s Republic of China

**Keywords:** Total polar compounds (TPC), Lipid metabolism, Oxidation stress, HepG2 cell

## Abstract

**Background:**

Recently, the harmful effects of frying oil on health have been gradually realized. However, as main components of frying oils, biochemical effects of total polar compounds (TPC) on a cellular level were underestimated.

**Methods:**

The effects of total polar compounds (TPC) in the frying oil on the lipid metabolism, oxidative stress and cytotoxicity of HepG2 cells were investigated through a series of biochemical methods, such as oil red staining, real-time polymerase chain reaction (RT-PCR), cell apoptosis and cell arrest.

**Results:**

Herein, we found that the survival rate of HepG2 cells treated with TPC decreased in a time and dose dependent manner, and thereby presented significant lipid deposition over the concentration of 0.5 mg/mL. TPC were also found to suppress the expression levels of *PPARα, CPT1 and ACOX*, elevate the expression level of *MTP* and cause the disorder of lipid metabolism. TPC ranged from 0 to 2 mg/mL could significantly elevate the amounts of reactive oxygen species (ROS) in HepG2 cells, and simultaneously increase the malondialdehyde (MDA) content from 21.21 ± 2.62 to 65.71 ± 4.20 μmol/mg of protein (*p* < 0.05) at 24 h. On the contrary, antioxidant enzymes superoxide dismutase (SOD), glutathione (GSH), and catalase (CAT) respectively decreased by 0.52-, 0.56- and 0.28-fold, when HepG2 cells were exposed to 2 mg/mL TPC for 24 h. In addition, TPC could at least partially induce the apoptosis of HepG2 cells, and the transition from G0/G1 to G2 phase in HepG2 cells was impeded.

**Conclusions:**

TPC could progressively cause lipid deposition, oxidative stress and cytotoxicity, providing the theoretical support for the detrimental health effects of TPC.

**Electronic supplementary material:**

The online version of this article (10.1186/s12944-019-0980-0) contains supplementary material, which is available to authorized users.

## Background

Total polar compounds (TPC) whose polarity are larger than those of triglycerides, are generated from peroxides and hydroperoxides during continuous frying, including short chain fatty acids, aldehydes, ketones, alcohol and nonvolatile products [1]. These are mainly responsible for quality deterioration of deep-fried food [2], which impart undesirable odor, unpleasant color and high viscosity to final products or fried oil [3]. Thus, it is speculated that TPC generated from the frying process of edible oil account for the unfavorable body health effects.

Recently, the harmful effects of frying oil on health have been gradually realized. It was reported that deep-fried oils and oxidized fat may take an unshirkable responsibility for inducing metabolic syndrome [4, 5]. For instance, frying oils could disturb liver function through inhibiting growth rate, promoting liver enlargement and attenuating detoxification enzymes related with defense mechanisms against in vivo lipid peroxidation [6, 7]. In addition, frying oil and its polar parts could potentially cause deformities in pregnant mice and perturbation of the vitamin A metabolic genes expression in fetal liver [8]. To our knowledge, however, the biochemical effects of TPC (the main components of frying oils) on a cellular level were underestimated.

Frying oil could result in the overloading of triglycerides, which further damage lipid constituents and cell function, and thus disturb lipid metabolism [7, 9, 10] which is closely correlated with the key genes involved in β-oxidation, such as *PPARα* (peroxisome proliferators-activated receptor alpha), *ACOX* (acyl-CoA oxidase) and *CPT-1* (carnitine palmitoyltransferase-1) [11–14]. Many researches demonstrated *MTP* (microsomal triglyceride transfer protein) was required for transporting triglyceride and assembling VLDL (very low density lipoproteins) in the liver, contributing to lipid metabolism [15, 16]. Also, the disorder of lipid metabolism occurred with excessive lipid accumulation and lipid peroxidation, leading to the imbalance of oxidative stress [17, 18] Zachary et.al [19] found that the dysregulation of *CPT* mediated by *PPARα* could promote the production of reactive oxygen species (ROS) in mitochondrion. Dysfunction of lipid metabolism could trigger oxidative stress through nuclear receptors [20]. On the another hand, oxidative stress might trigger the occurrence of cell apoptosis and cycle arrest [21]. Interestingly, both cell apoptosis and cycle arrest on behalf of cytotoxicity were regarded as prominent pathogenesis of liver diseases [22] demonstrating the progressive relationship between oxidative stress and cytotoxicity. Accordingly, to better understand the biochemical influence of frying oil containing TPC, exploring the changes of lipid metabolism, oxidative stress and cytotoxicity with the addition of TPC is indispensable.

Taken together, we reckoned that the biochemical effects of TPC originate from dysregulation of lipid metabolism, which further lead to oxidative stress and thereby trigger cell apoptosis and cycle arrest. Our previous study has proved that TPC could affect the lipid metabolism and liver functions of mice [7] while the biochemical effects of TPC on a cellular level were inadequate and nonsystematic. Thus, to confirm our hypothesis, we assessed the physiological changes of lipid metabolism, the level of oxidative stress and the cytotoxicity in HepG2 cells.

## Materials and methods

### Materials

Peanut oil without antioxidant was supplied by Dehe Food Technology Co., Ltd. (Wuxi, China). Chicken legs (Tyson Foods Inc.) were purchased at a local supermarket. The HepG2 cell, an immortalized human hepatoma cell line, was bought from the Institute of Biochemistry and Cell Biology, Shanghai Institutes for Biological Sciences (SIBS) (Shanghai, China). Minimum essential medium (MEM), fetal bovine serum (FBS), trypsin and other cell culture materials were purchased from Gibco BRL, Life Technologies (Carlsbad, CA, USA). Cell counting kit-8 (CCK-8), triglyceride (TG), malondialdehyde (MDA), catalase (CAT), superoxide dismutase (SOD) and total glutathione quantification (GSH) assay kits were all obtained from Beyotime Biotechnology Co., Ltd. (Shanghai, China). The reactive oxygen species (ROS) and bicinchoninic acid (BCA) protein assay kits were purchased from Thermo Fisher Scientific (Waltham, MA, USA). The annexin V-fluorescein isothiocyanate (FITC) apoptosis detection kit and cell cycle analysis kit were all purchased from Gibco BRL, Life Technologies (Carlsbad, CA, USA). UNIQ-10 column total RNA extraction kit, avian myeloblastosis virus reverse transcriptase kit and 2 × SG fast qPCR master mix kit were purchased from Sangon Biotech Co., Ltd. (Shanghai, China). All chemicals and reagents were of analytical grade or higher.

### Frying process

Peanut oil (7 L) was placed in an 8-L capacity bench-top electric fryer (Shanghai Precision & Scientific Instrument, int., Shanghai, China) and maintained at 180 ± 2 °C. Four raw chicken legs (around 480 g) were put in electric fryer every 1 h to simulate normal scenes of fried food. No replenishment of fresh oil was topped up during the frying process. Frying experiment was carried out for 40 h. Peanut oil samples were collected and stored in the dark at − 20 °C for further chemical and physical analysis. Three replications of 40-h deep frying trials were performed.

### Total polar components (TPC)

Peanut oil of 40-h deep frying was ready for the preparation of TPC, which was built on the silica gel column chromatography. In order to more effectively separate TPC, real-time monitoring was conducted by using thin-layer chromatography. The silica columns (Shanghai Shendi Glass Instrument Co., Ltd., Shanghai, China) with silica gel (40 g; model specifications: 200–300 meshes per inch) were washed with the solvent A (petroleum ether: ethyl ether = 87:13, *v*/v). Then the peanut oil sample was dissolved in solvent A for pouring into the column. The TPC were collected with solvent B (ethyl ether) and solvent C (methanol) after extraction of the non-polar components with solvent A. Then, the TPC separately dissolved in solvent B and solvent C was subsequently conducted to rotary evaporation (40 °C) and vacuum drying (40 °C, 0.01 MPa).

### Cell culture

HepG2 cells were gradually grown in MEM supplemented with 10% fetal bovine serum (FBS) and 1% penicillin/streptomycin at 37 °C in a humidified atmosphere containing 5% of CO_2_. After incubation, cells were rinsed with phosphate-buffered saline buffer (PBS, pH 7.4), following the addition of 1 mL trypsin (0.02% EDTA, 0.25% trypsin) for 2 min to digest cells. MEM with 10% fetal bovine serum (FBS) was added to terminate the digestion. Then cells were collected by centrifuging at 800 rpm for 5 min. All the cells were in the logarithmic phase of the experiments.

### Detection of cell viability

The viability ratio of HepG2 cells after treating with TPC was assessed using CCK-8 colorimetric assay. Briefly, HepG2 cells were planted in a 96-well plate (5 × 10^3^cells/well). After 12, 24 and 48 h treatment with TPC at the concentrations of 0–6 mg/mL, 10 μL CCK-8 solution was added into the wells and the plates were further incubated at 37 °C and 5% of CO_2_ for 4 h to produce WST-8 formazan orange dye. Then, the absorbance was detected with a microplate reader (BioTek Instruments, Winooski, VT, USA) at 450 nm. The cell viability was calculated as follows:

$$ \mathrm{Viability}\ \mathrm{rate}=\frac{\mathrm{ODsample}}{\mathrm{ODcontrol}}\times 100\% $$

### Evaluation of hepatic TG levels

TG accumulation was determined by Oil Red O staining and TG kit. HepG2 cells were treated with TPC at the concentrations from 0 to 2 mg/mL for 24 h. For Oil Red O Staining, HepG2 cells were washed by PBS (pH 7.4) before fixed with formalin for 30 min. Then Oil Red O was used to stain cells. As for the TG content, total samples were collected and measured by TG kit after homogenizing and rupturing HepG2 cells. Protein levels in cells were analyzed by using BCA protein assay kit to standardize the amount of TG.

### Determination of intracellular ROS

HepG2 cells were collected after 0, 0.1, 0.5, 1 and 2 mg/mL TPC-treatment for 12, 24 and 48 h. Cellular ROS were detected by using ROS assay kit according to the manufacturer’s protocol. Flow cytometer (BD FACSCalibur, San Jose, CA, USA) was used at the excitation and emission wavelengths of 488 and 525 nm for quantitative detection. The relative level of ROS was measured by detecting the fluorescence of fluorescent-oxidized derivative of dichlorofluorescein (DCF).

### Measurement of MDA content and antioxidant enzymes activity

After TPC treatment (0–2 mg/mL) for 12, 24 and 48 h, HepG2 cells were lysed by western and IP cell lysates, homogenized and centrifuged at 5000 g for 10 min at 4 °C. The supernatant was collected and added into 96-well plates for the following measurements. The total MDA, SOD, CAT and GSH activities were determined by using assay kits according to manufacturer’s protocols. The protein content of the supernatant was determined by using the BCA assay kit to standardize the data.

### Detection of cell cycle

HepG2 cells were incubated in 6 cm-plates (1 × 10^5^ cells/mL) with different TPC concentrations (0–2 mg/mL) for 12, 24 and 48 h. Subsequently, cells were collected with MEM and rinsed with 4 °C PBS (pH 7.4). Then, 1 mL of 70% ethanol was added in cells for 24 h. After rinsing with 4 °C PBS (pH 7.4), the cells were well mixed with 0.5 mL of the propidium iodide (PI) staining solution. Cell cycle distribution was detected by flow cytometry (BD FACSCalibur, San Jose, CA, USA). The proportions of cell distribution at different phases during were analyzed through ModiFit LT software (Verity Software, Topsham, ME, USA).

### Evaluation of cell apoptosis

Annexin V-FITC and propidium iodide (PI) were used to assess the apoptosis proportion of HepG2 cells. HepG2 cells were treated with 0, 0.1, 0.5, 1 and 2 mg/mL TPC for 12, 24 and 48 h. After collecting cells, samples were dispersed in 4 °C PBS (pH 7.4) and diluted to concentration of 5 × 10^5^ cells/mL. Then, 300 μL of 1 × binding buffer, 5 μL of annexin V-FITC and 5 μL of propidium iodide (PI) were successively added into the cells. After incubating at 37 °C and 5% of CO_2_ for 15 min, the cell apoptosis rate was assessed by flow cytometry (BD FACSCalibur, San Jose, CA, USA).

### RNA isolation and real-time polymerase chain reaction (RT-PCR)

Total RNA was isolated with UNIQ-10 column total RNA extraction kit. The purity and quantity of the obtained total RNA samples were measured by ultra-micro spectrophotometer (Thermo, Waltham, MA, USA). Reverse-transcribed RNA was performed by using an avian myeloblastosisvirus reverse transcriptase kit. The reverse transcription was conducted in conditions of 42 °C for 60 min. Quantitative RT-PCR was performed by using 2 × SG fast qPCR master mix kit and all experiments were done in triplicates. The primers sequences are listed in Table 1. *β-actin* was used as an internal reference and the relative gene expression was finally calculated by fold induction (2^-ΔΔCt^) [23].

**Table 1:** Primer sequences used for RT-PCR

Gene	Accession number	Forward primer (5′- 3′)	Reverse primer (5′- 3′)
*β-actin*	NM_004882	AAGGAGCCCCACGAGAAAAAT	ACCGAACTTGCATTGATTCCAG
*PPARα*	NM_005036	ATGGTGGACACGGAAAGCC	CGATGGATTGCGAAATCTCTTGG
*CPT1*	NM_004377	GCGCCCCTTGTTGGATGAT	CCACCATGACTTGAGCACCAG
*ACOX*	NM_003500	GCACCCCGACATAGAGAGC	CTGCGGAGTGCAGTGTTCT
*MTP*	NM_018109	GGGCTCTTGACCCGTTTGAA	TCGTCTCTCCTAAGGTCTTTGG

### Statistical analysis

Data was showed as the mean ± standard error of mean (SEM). The statistical analysis was performed by one-way analysis of variance (ANOVA) and Duncan’s multiple range test using SPSS package. The *p* < 0.05 was considered significant.

## Results

### Effect of TPC on lipid accumulation in HepG2 cells

To investigate the biohazard of TPC, we conducted the cell viability assay and found that more than half of HepG2 cells were death over the concentration of 2 mg/mL TPC in 48 h (Additional file 1: Figure S1). Thus, HepG2 cells treated with 0, 0.1, 0.5, 1 or 2 mg/mL of TPC were stained with Oil Red O to observe their histological morphology.

**Fig. 1:**
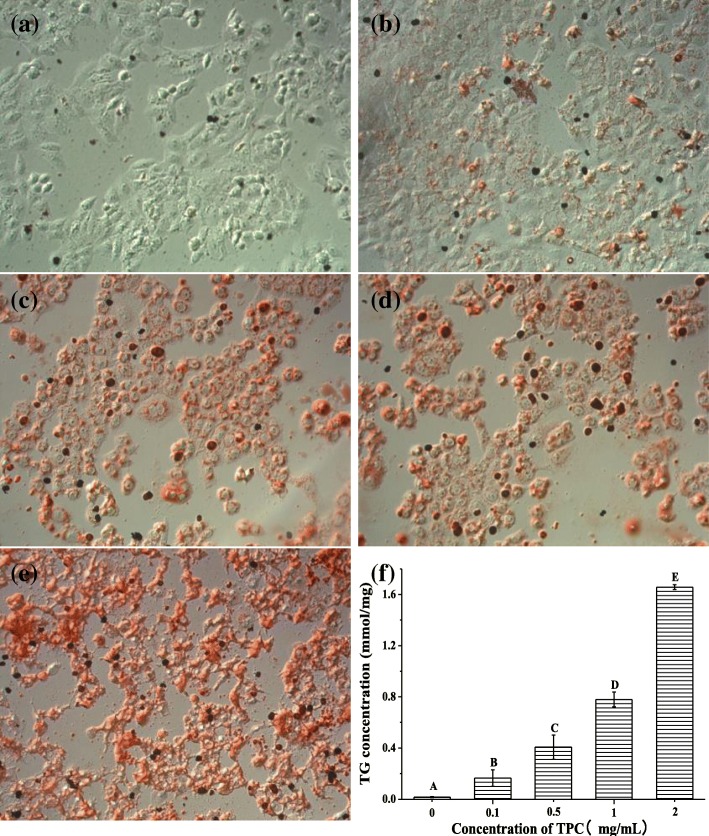
The level of lipid accumulation. Oil red staining of HepG2 cells treated with TPC at the concentration of (**a**) 0 mg/mL (control group), (**b**) 0.1 mg/mL, (**c**) 0.5 mg/mL, (**d**) 1 mg/mL and (**e**) 2 mg/mL; the content of triglyceride after treatment with different concentrations of TPC (**f**) for 24 h. Means with different letters (A, B, C, D, E) were significantly different from one another by Duncan’s multiple-range test (*p* < 0.05)

As described in Fig. 1, a dose-dependent manner of lipid accumulation was observed in HepG2 cells. Few cells in control group were stained with the red color, whereas a hint of red presented in the cells treated with 0.1 mg/mL TPC (Fig. 1a and b). As the increasing concentrations of TPC, the proportion of cells stained with red color augmented significantly (Fig. 1c, d and e). After exposure to 2 mg/mL of TPC, specifically, almost all of the cells were filled with Oil Red O, demonstrating the lipid accumulation and cell deformation. To further confirm this, triglyceride contents in the supernatant of HepG2 cells treated with TPC were measured to assess the state of lipid deposition quantitatively. As shown in Fig. 1f, triglyceride in the HepG2 cells treated with 0.1 mg/mL of TPC was 0.17 ± 0.06 mmol/mg of protein, which was significantly higher than that of control group (0.02 ± 0.00 mmol/mg of protein; *p* < 0.05). An obvious increasing trend in intracellular triglycerides was achieved as the growing concentrations of TPC, reaching 1.66 ± 0.02 mmol/mg of protein in the concentration of 2 mg/mL (*p* < 0.05). Therefore, these results demonstrated a dose-dependent increase of lipid accumulation in HepG2 cells treated with TPC.

**Fig. 2:**
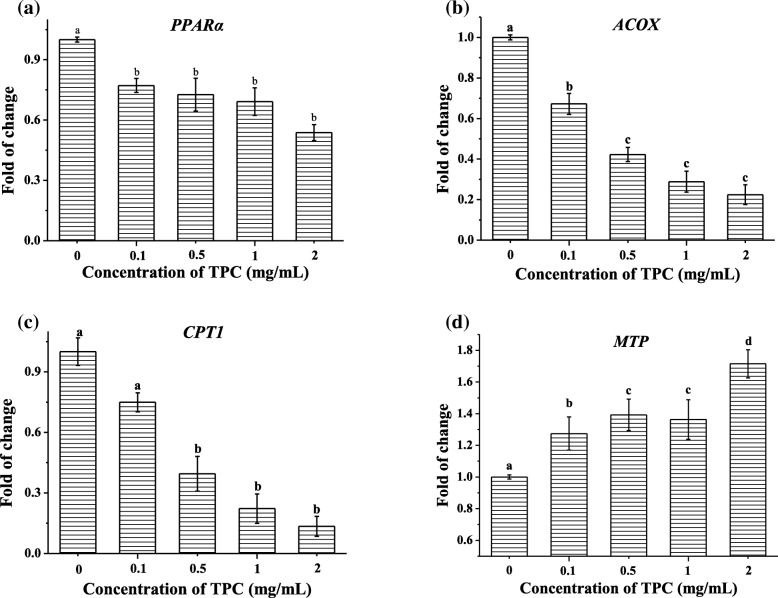
The expression levels of (**a**) *PPARα*, (**b**) *ACOX*, (**c**) *CPT1* and (**d**) *MTP*. Cells were treated with 0.1 mg/mL, 0.5 mg/mL, 1 mg/mL, 2 mg/mL, prior to control group (0 mg/mL) for 24 h. Means with different letters (A, B, C, D) were significantly different from one another by Duncan’s multiple-range test (*p* < 0.05)

Overloading of lipid in cells might disturb lipid metabolism. Thus, the effects of TPC on the genes involved in lipid metabolism were evaluated using RT-PCR. As depicted in Fig. 2a, after TPC stimulation, the expression levels of *PPARα* were significantly dropped to 0.77 ± 0.03 fold (*p* < 0.05) at 0.1 mg/mL of TPC compared with the control group. As shown in Fig. 2b and c, the expressions levels of *ACOX* and *CPT1* were consistently inhibited with TPC concentrations increasing. The inhibition rates of *ACOX* and *CPT1* were respectively from 0.67 ± 0.05 to 0.22 ± 0.05 fold (*p* < 0.05) and from 0.75 ± 0.05 to 0.13 ± 0.05 fold (*p* < 0.05), when the concentration of TPC increased from 0.1 to 2 mg/mL. Figure 2d showed that the expression level of *MTP* was increased by 1.72 ± 0.09 fold (*p* < 0.05) after TPC incubation from 0 mg/mL to 2 mg/mL.

### TPC induced ROS production in HepG2 cells

**Fig. 3:**
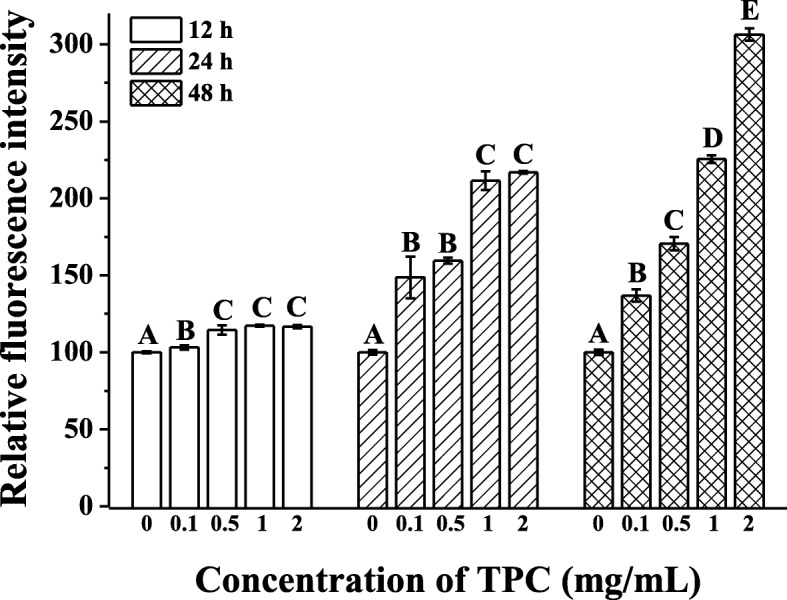
Effects of TPC on intracellular ROS levels of HepG2 cells. Cells were treated with 0.1 mg/mL, 0.5 mg/mL, 1 mg/mL, 2 mg/mL, prior to control group (0 mg/mL) for 12, 24 and 48 h. Means with different letters (A, B, C, D, E) were significantly different from one another by Duncan’s multiple-range test (*p* < 0.05)

It was well acknowledged that lipid accumulation could induce the generation of ROS [21]. Thus, ROS production in TPC-treated HepG2 cells was measured to evaluate the consequence of lipid deposition. Figure 3 depicted that the fluorescence intensity remarkably elevated with the increasing concentrations of TPC for 12, 24 and 48 h, suggesting the augment of intracellular ROS levels. Compared with control group, accumulation of relative fluorescence intensity rose about 1.17 ± 0.01 folds (*p* < 0.05) in 12 h, 2.17 ± 0.01 folds (*p* < 0.05) in 24 h and 3.06 ± 0.04 folds in 48 h (*p* < 0.05) with the increasing of TPC concentrations. The results revealed that the accumulation of ROS increased significantly with the 2 mg/mL TPC as time increased.

### TPC induced oxidative stress

**Fig. 4:**
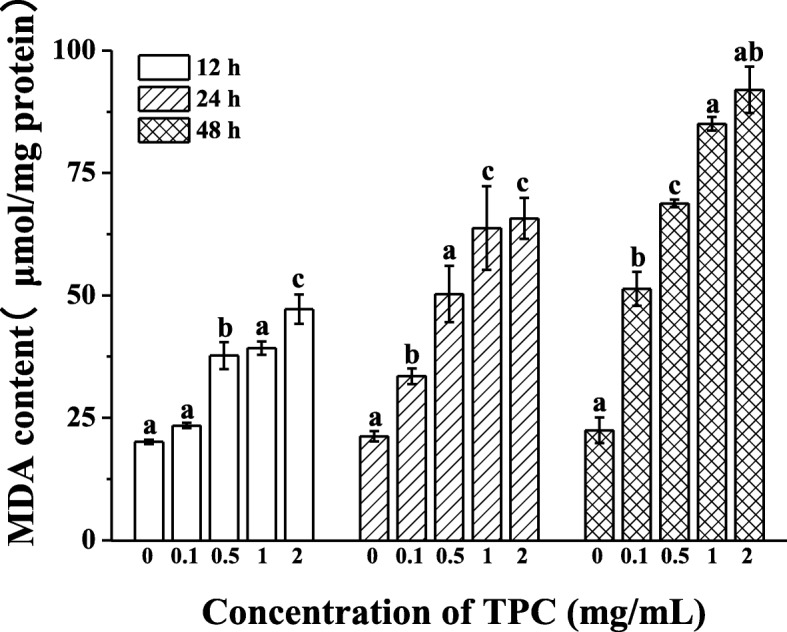
TPC induced MDA formation in HepG2 cells. Cells were treated with 0.1 mg/mL, 0.5 mg/mL, 1 mg/mL, 2 mg/mL, prior to control group (0 mg/mL) for 12, 24 and 48 h. Means with different letters (A, B, C) were significantly different from one another by Duncan’s multiple-range test (*p* < 0.05)

The elevated ROS production disturbed oxidative stress system, resulting in lipid peroxidation [24]. To confirm this, the lipid peroxidation in HepG2 cells was assessed by determining MDA levels. As shown in Fig. 4, the content of MDA was visibly increased after TPC stimulation for 12 h, which was from 20.09 ± 0.46 μmol/mg of protein to 47.18 ± 3.02 μmol/mg of protein (*p* < 0.05). And at the same time, the same trends were highly evidently when treated with TPC for 24 h (from 21.21 ± 1.06 μmol/mg of protein to 65.71 ± 4.20 μmol/mg of protein) (*p* < 0.05) and 48 h (from 22.46 ± 2.62 μmol/mg of protein to 91.96 ± 4.71 μmol/mg of protein) (*p* < 0.05).

**Fig. 5:**
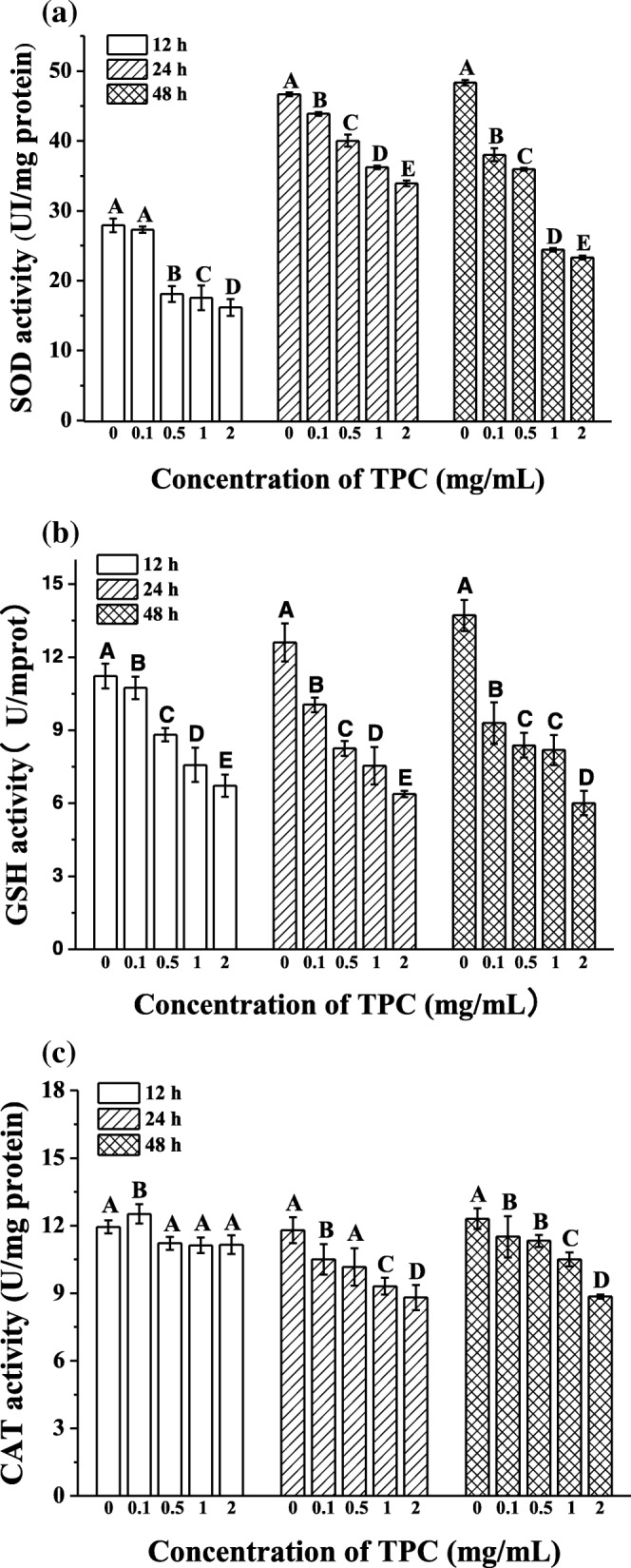
Effects of TPC on the activities of (**a**) SOD, (**b**) CAT and (**c**) GSH. Cells were treated with 0.1 mg/mL, 0.5 mg/mL, 1 mg/mL, 2 mg/mL, prior to control group (0 mg/mL) for 12, 24 and 48 h. Means with different letters (A, B, C, D, E) were significantly different from one another by Duncan’s multiple-range test (*p* < 0.05)

The decline of oxidative stress-induced oxidoreductaseis activity was considered to be a crucial link in the process of liver diseases. To further investigate the effects of oxidative stress on TPC-induced HepG2 cells, we tested the activities of antioxidative enzymes. The results showed that activity of SOD, GSH and CAT in HepG2 cells was decreased obviously and showed a dose-effect relationship, presenting the declination of 0.52-, 0.56- and 0.28-fold, respectively, after exposed to 2 mg/mL TPC for 48 h. As shown in Fig. 5a, SOD activity treated with TPC for 12, 24 and 48 h was dropped by 11.73 ± 1.20, 12.75 ± 0.38 and 25.02 ± 0.28 UI/mg protein, respectively, in comparison of control group (*p* < 0.05). A significant depressed tendency was showed in GSH activity (Fig. 5b), which reduced by 4.50 ± 0.45 UI/mg protein (*p* < 0.05) at 12 h. However, CAT activity had different tendency at 12 h, and it went through a transient increase at a concentration of 0.1 mg/mL before continuous decreasing with increased concentration (*p* < 0.05; Fig. 5c).

### TPC induced apoptosis

**Fig. 6:**
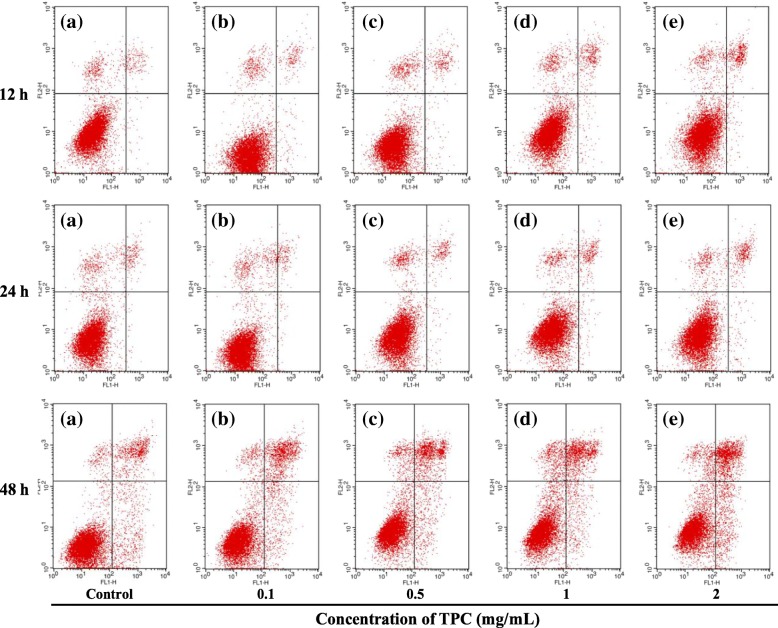
Effects of TPC on cell apoptosis in HepG2 cells. Flow cytometric analysis for apoptosis induction of HepG2 cells treated with TPC at the concentrations of (**a**) 0 mg/mL, (**b**) 0.1 mg/mL, (**c**) 0.5 mg/mL, (**d**) 1 mg/mL, and (**e**) 2 mg/mL for 12, 24 and 48 h

Annexin V-FITC/PI staining was used to detect apoptosis of TPC on HepG2 cells and study the mechanism of lipid peroxidation-induced apoptosis. As Fig. 6 and Table 2 showed, the percentage of apoptotic cells increased with the increasing concentrations of TPC. Specifically, the proportion of apoptotic cells increased to 9.99 and 14.53%, respectively, after 12 and 24 h treatment of 2 mg/mL TPC. When exposed to 2 mg/mL TPC for 48 h, the amount of apoptotic cells increased by 20.23% compared to the control group. These results further indicated that TPC-induced degradation in cell viability was responsible for the decreasing of apoptosis.

**Table 2:** Apoptosis rate of HepG2 cells treated with TPC

Time (h) Concentration (mg/mL)	12 Apoptosis rate (%)	24 Apoptosis rate (%)	48 Apoptosis rate (%)
0	5.85	6.77	9.76
0.1	6.46	7.4	21.99
0.5	7.7	7.63	23.38
1	9.18	8.02	24.25
2	9.99	14.53	29.99

### Effects of TPC on cell cycle in HepG2 cells

**Fig. 7:**
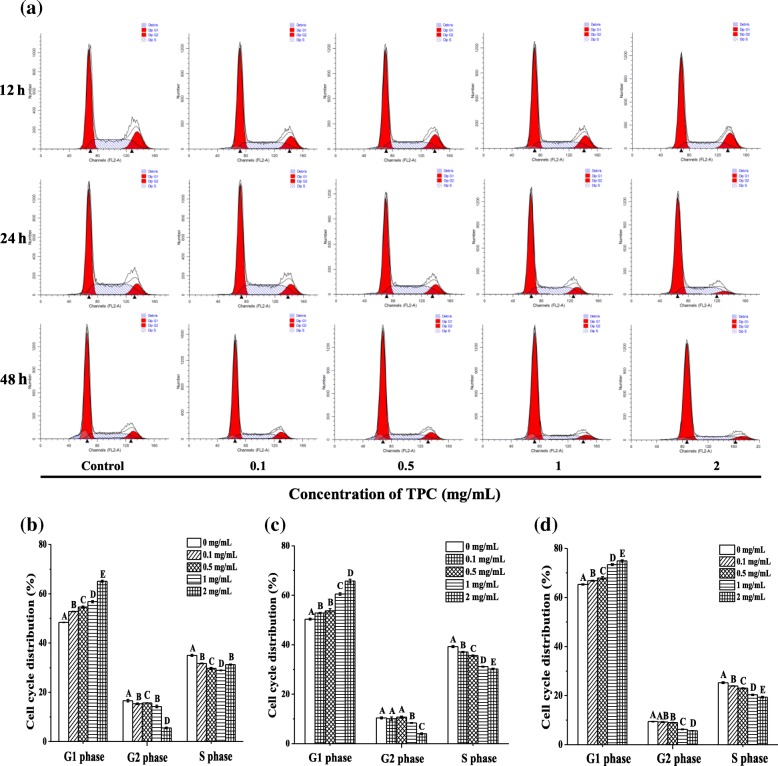
Effects of TPC on cell cycle in HepG2 cells. The cell cycle diagram of HepG2 cells treated with TPC (**a**) and the proportion of the HepG2 phase of G1, G2 and S in cells incubated with TPC for 12 h (**b**), 24 h (**c**) and 48 h (**d**). Means with different letters (A, B, C) were significantly different from one another by Duncan’s multiple-range test (*p* < 0.05)

Cell cycle is another important basis for detecting cytotoxicity. To investigate the cell cycle distribution, HepG2 cells were stained with PI/RNase staining buffer by flow cytometry. As shown in Fig. 7, with the increasing of TPC concentrations (0, 0.1, 0.5, 1, 2 mg/mL), the number of cells treated with TPC for 12 h in G0/G1 phase obviously increased (from 48.41 to 65.15%), coinciding to these of 24 h (from 50.34 to 65.77%) and 48 h (from 65.30 to 74.88%). However, HepG2 cells in G2 phase decreased by 11.04, 6.41 and 3.68% after TPC treatment for 12, 24 and 48 h. It revealed that TPC could prevent cell transition from G0/G1 to G2 phase, indicating TPC could destroy the cell proliferation.

## Discussion

Over the past several decades, the potential health risk of deep-frying food has attracted more and more attention. The common indicator to evaluate the quality of deep-frying food is TPC, whereas its biochemical influence on a cellular level is still underestimated. Herein, we explored the status about the lipid metabolism and oxidative stress of HepG2 cells under the treatment of TPC. Cell apoptosis and cycle arrest were sequentially investigated to further explain the action of TPC on HepG2 cells.

Excessive intake of free fatty acids by HepG2 cells should active lipogenesis-related enzymes, which further promote the production of intracellular lipid [25, 26]. We found TPC could significantly induce lipid accumulation in HepG2 cells and reduce the cell survival, corresponding to the behavior of oxidized oil [27]. Accordingly, we reasoned that TPC would cause lipid accumulation and subsequently affect lipid metabolism, such as metabolic genes and lipid transporter gene. As the regulated genes of lipolysis, *PPARα*, *ACOX* and *CPT1* exert important roles on keeping lipid away from deposition and steatosis [28–31]. These three investigated genes in HepG2 cells were remarkedly down-regluated after the exposure to TPC, providing support for the view that TPC could (at least partly) lead to excessive fat intake. This might be due to the inhibition of β-oxidation in mitochondria and the silence of peroxisomes caused by the percolating TPC. What’s more, elevated MTP levels further evidenced the lipid metabolism disorders of HepG2 cell triggered initially by TPC, in consistent with previous research [32, 33]. The block of VLDL (very low density lipoprotein) formation and lipoprotein assembly caused by *MTP* inhibition might lead to lipid deposition [33–35]. However, there are still some limitations in this present study. The precise mechanism involved in lipid content increasing in the HepG2 cells is still not fully elucidated. The important role of lipid metabolism regulation in lipid accumulation could be further studied through lipogenesis or agonists and inhibitors.

Intriguingly, *PPARα* and *CPT1* are also bound up with oxidative stress and the production of ROS [36–38]. We thus imagined that the occurrence of lipid accumulation would evoke the exceedance of ROS production and bring about the imbalance of oxidative-antioxidant system. We found the elevated ROS and MDA content as well as the reduction of SOD, CAT and GSH activity in TPC-treated HepG2 cell, which was in agreement with other reports [39, 40]. Consequently, the accumulation of triglycerides increased the generation of ROS and induced the lipid peroxidation. As Bakker et al. [41] reported, deposition of triglycerides might promote the production of O_2_^−^ in the mitochondrial electron transport chain, and then the accumulation of free radicals would cause lipid peroxidation.

Metabolic deregulations and oxidative stress have a crucial role in hepatocyte damage and apoptosis [21]. By detection of apoptosis and cell cycle, TPC was confirmed to induce HepG2 cells apoptosis and to prevent HepG2 cells transition from G0/G1 to G2 phase. This result was in consist with our previous study about frying palm oil, where showed the different parts of TPC could inhibit the proliferation of HepG2 cells, cause elevated cell apoptosis and abnormal cycle arrest [42]. Oxidative stress could result in the damage of cellular biomacromolecules like DNA, further leading to mitochondrial dysfunction and cell apoptosis [43]. ROS is closely linked with DNA damage, and the latter will trap cell into gap1 (G1), DNA synthesis (S), or in gap2/mitosis (G2/M) phase [44, 45], in agreement with our study (data are not shown). Therefore, the excessive ROS production or oxidative stress in TPC-treated cells might damage biomacromolecules, induce cell apoptosis, and make the cells stagnate in G0/G1phase.

**Fig. 8:**
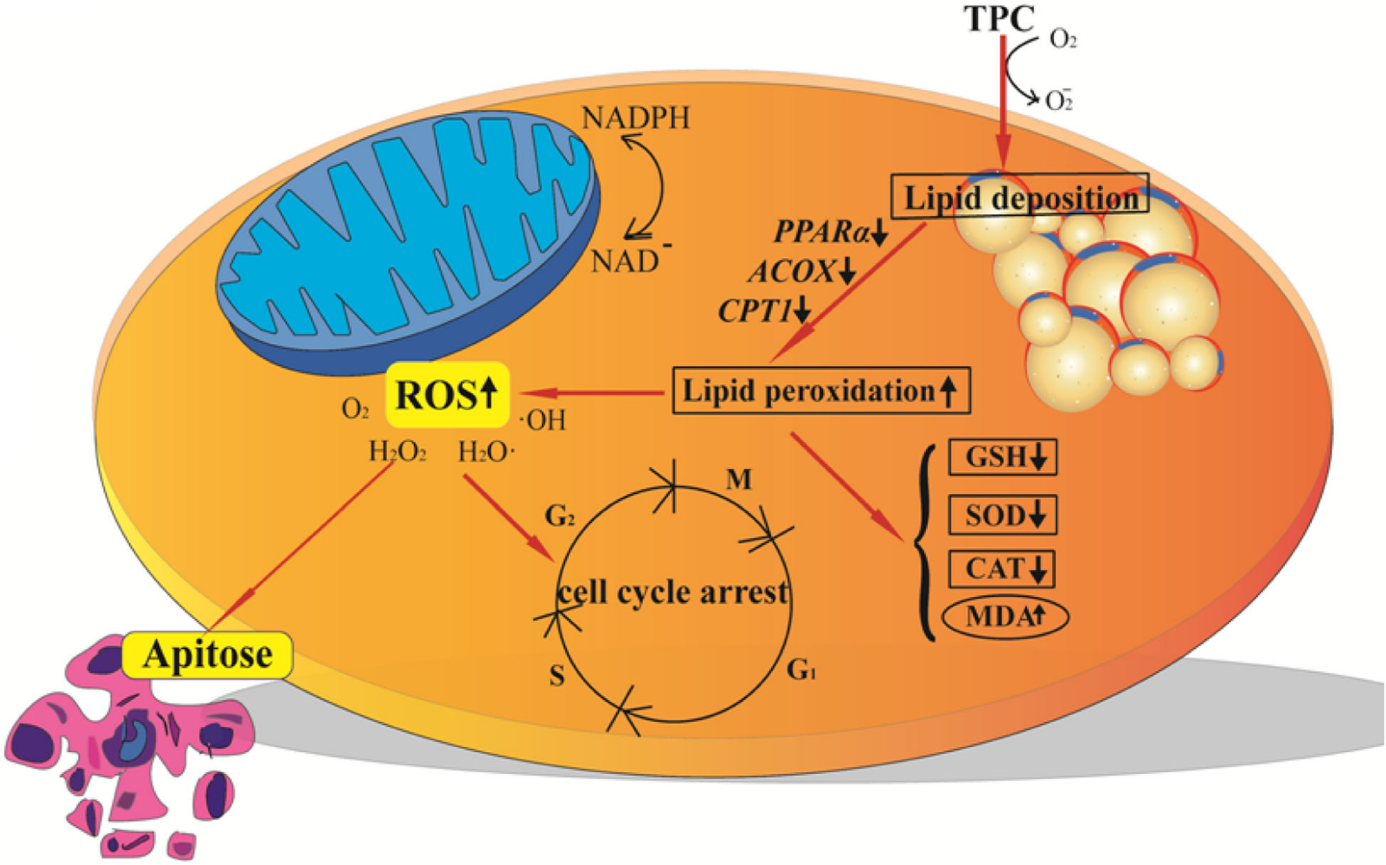
The proposed action of TPC on HepG2 cell

Taken all together, we primitively outline the behavior of TPC and its mode of action. As shown in Fig. 8, TPC over a certain dose (0.1 mg/ml) is potential to cause lipid accumulation, evoke the occurrence of lipid metabolic dysregulation and lipid peroxidation. Simultaneously, the increasing production of ROS and MDA exacerbate lipid oxidation and lead to oxidative damage, as well as the decreasing activities of SOD, CAT and GSH. Cells trapped in the status oxidative stress may damage the cell function and even promote cell apoptosis. Therefore, TPC lead to lipid deposition, and further make cells undergo the oxidative stress, which triggers cell apoptosis and cell cycle arrest.

## Conclusion

In conclusion, our results demonstrated that TPC could reduce the HepG2 cell survival and induce lipid accumulation in a dose-dependent manner, accompanying with the down-regulation of metabolic genes *PPARα, ACOX* and *CPT1* and up-regulation of lipid transporter gene *MTP*. Oxidative stress in HepG2 cell was also confirmed by means of increasing the production of ROS and MDA but decreasing the activities of antioxidant enzymes (SOD, CAT and GSH). Moreover, TPC was found to induce HepG2 cells apoptosis and to prevent HepG2 cells transition from G0/G1 to G2 phase, demonstrating the occurrence of cytotoxicity. Thus, TPC could cause lipid accumulation and trigger the disorder of lipid metabolism, oxidative stress and cytotoxicity, supporting for detrimental health influence of TPC.

## Additional files

Additional file 1: **Figure S1.** Cell viability of HepG2 cells. (DOCX 92 kb)

## Abbreviations

CAT: Catalase
DCFH-DA 2,: 7-Dichlorodihydrofluorescein diacetate
FITC: Fluorescein isothiocyanate
GPX: Glutathione peroxidase
MDA: Malondialdehyde
PI: Propidium iodide
ROS: Reactive oxygene species
RT-PCR: Real-time polymerase chain reaction
SOD: Superoxide dismutase
TG: Triglyceride
TPC: Total polar compounds
