# Supplementary material for: Influence of total polar compounds on lipid metabolism, oxidative stress and cytotoxicity in HepG2 cells
Source: Lipids Health Dis. 2019 Feb 1;18:37. doi: 10.1186/s12944-019-0980-0 (PMC6359786; doi:10.1186/s12944-019-0980-0)
Supplement: Supplementary file 1 — Figure S1. Cell viability of HepG2 cells. (DOCX 92 kb) [file 12944_2019_980_MOESM1_ESM.docx]

**Fig. S1**

**Fig. S1 Cell viability of HepG2 cells. TPC induced concentration-dependent decrease in cellular proliferation of treated cells, in relation to control were examined via CCK-8 assay at dilutions of TPC (0.1 mg/mL, 0.5 mg/mL, 1 mg/mL, 1.5 mg/mL, 2 mg/mL, 3 mg/mL, 4 mg/mL, 5 mg/mL and 6 mg/mL) for 12 h, 24 h, 48 h.** Means with different letters (A, B, C, D, E, F) were significantly different from one another by Duncan’s multiple-range test (*p* < 0.05).
